# Supplementary material for: Circulating miR-320a as a Predictive Biomarker for Left Ventricular Remodelling in STEMI Patients Undergoing Primary Percutaneous Coronary Intervention
Source: J Clin Med. 2020 Apr 8;9(4):1051. doi: 10.3390/jcm9041051 (PMC7230612; doi:10.3390/jcm9041051)
Supplement: Supplementary file 1 [file jcm-09-01051-s001.pdf]

Circulating miR-320a as a Potential Biomarker for Left Ventricular Remodeling in STEMI Patients Undergoing Primary Percutaneous Coronary Intervention

Galeano-Otero I, et al.

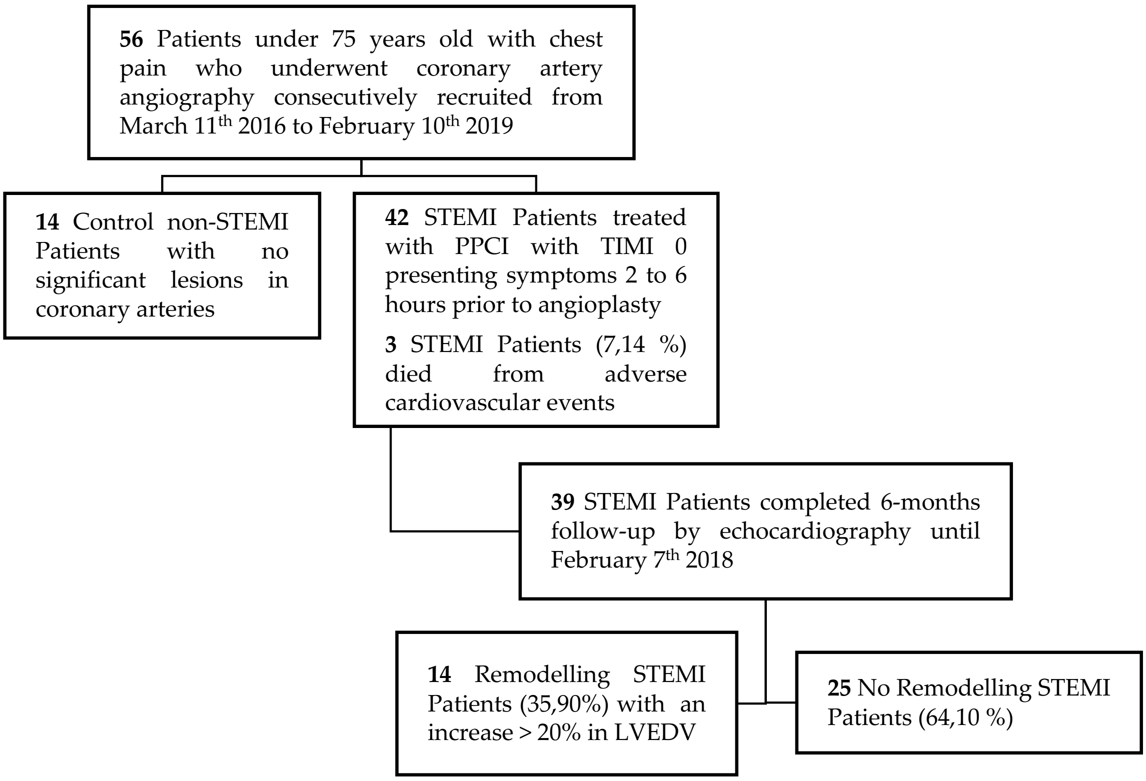

Online Figure S1. STROBE diagram of the subjects and the study design. STEMI: ST-segment-elevation myocardial infarction; LVEDV: left ventricular end-diastolic volume.

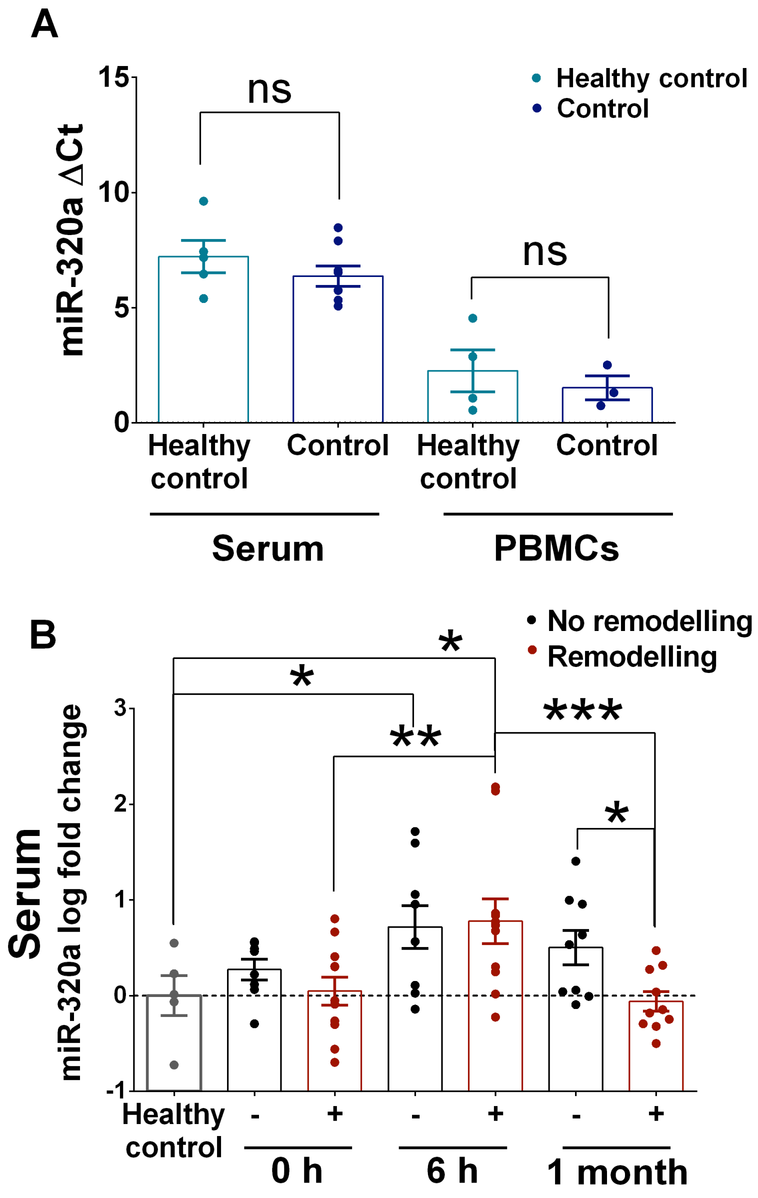

Online Figure S2. miR-320a expression in serum and PBMCs of STEMI patients compared to healthy controls. (A) Graph showing the expression of miR-320a in control groups (n = 8) and healthy volunteers (n = 5) in serum and PBMCs. The expression of miR-320a was assessed in serum (B) 0 hour, 6 hours and 1 month after PPCI in STEMI patients who developed LVAR (red bar, n = 11) or not (black bar, n = 9) compared to the miR-320a levels in healthy volunteers. The values in the graphs represent the fold change in logarithmic scale for each miRNA relative to the controls. Data are the means ± SEM. Significance is indicated by (*) for p<0.05, (**) for p<0.01, and (***) for p<0.001). Ordinary one-way Anova with multiples comparisons using T test without correct (Fisher's LSD test) was performed.

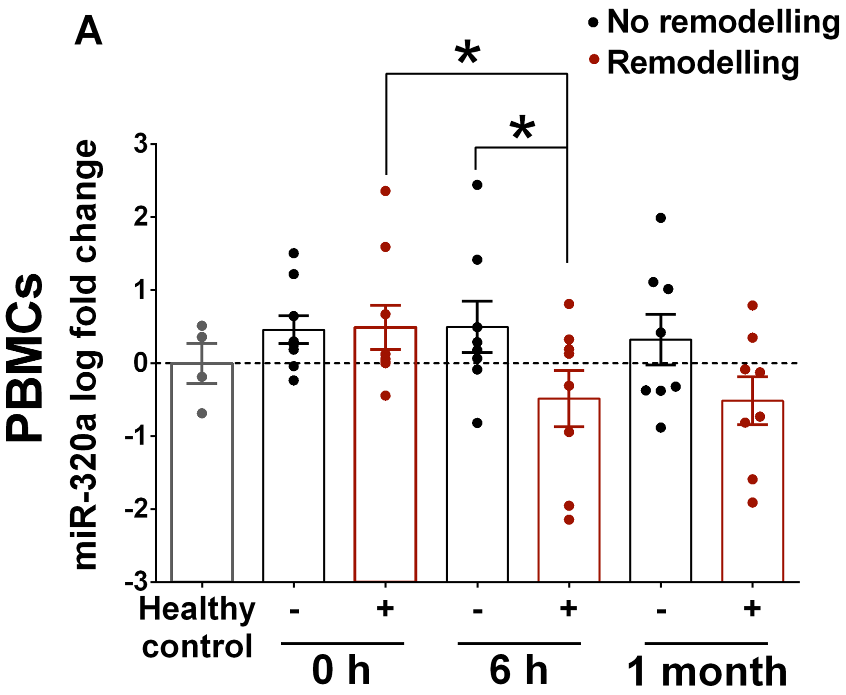

Online Figure S3. miR-320a expression in PBMCs of STEMI patients compared to healthy controls. Graph showing the expression of miR-320a in PBMCs 0 hour, 6 hours and 1 month after PPCI in STEMI patients who developed LVAR (red bar, n = 11) or not (black bar, n = 9) compared to the miR-320a levels in healthy volunteers. The values in the graphs represent the fold change in logarithmic scale for each miRNA relative to the controls. Data are the means ± SEM. Significance is indicated by (*) for p<0.05. Ordinary one-way Anova with multiples comparisons using T test without correct (Fisher's LSD test) was performed.

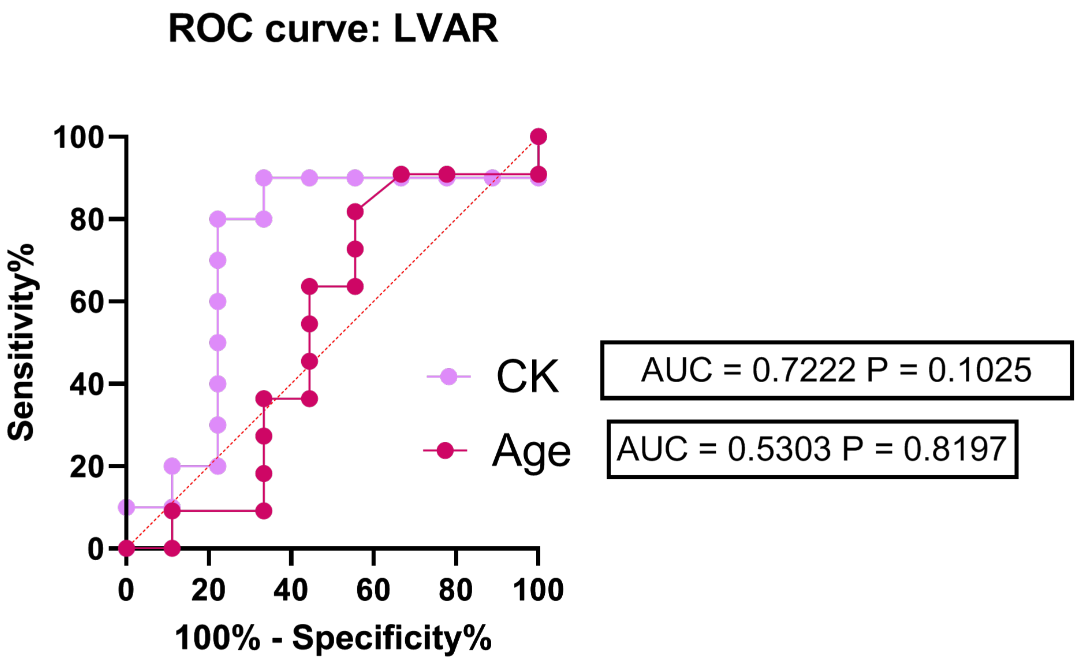

Online Figure S4. Receiver-operating characteristic (ROC) curves comparing sensitivity and specificity of Creatine Kinase and Age in predicting left ventricular adverse remodelling (LVAR). Values are given in the graph. AUC is for "area under the curve".
